# Mutations in the Human AAA^+^ Chaperone p97 and Related Diseases

**DOI:** 10.3389/fmolb.2016.00079

**Published:** 2016-12-01

**Authors:** Wai Kwan Tang, Di Xia

**Affiliations:** Laboratory of Cell Biology, Center for Cancer Research, National Cancer Institute, National Institutes of Health Bethesda, MD, USA

**Keywords:** VCP/p97, structure and function, mutations, conformational changes, multisystem diseases

## Abstract

A number of neurodegenerative diseases have been linked to mutations in the human protein p97, an abundant cytosolic AAA^+^ (ATPase associated with various cellular activities) ATPase, that functions in a large number of cellular pathways. With the assistance of a variety of cofactors and adaptor proteins, p97 couples the energy of ATP hydrolysis to conformational changes that are necessary for its function. Disease-linked mutations, which are found at the interface between two main domains of p97, have been shown to alter the function of the protein, although the pathogenic mutations do not appear to alter the structure of individual subunit of p97 or the formation of the hexameric biological unit. While exactly how pathogenic mutations alter the cellular function of p97 remains unknown, functional, biochemical and structural differences between wild-type and pathogenic mutants of p97 are being identified. Here, we summarize recent progress in the study of p97 pathogenic mutants.

## P97 associated diseases

### Multisystem proteinopathy (MSP)

**Figure 1:**
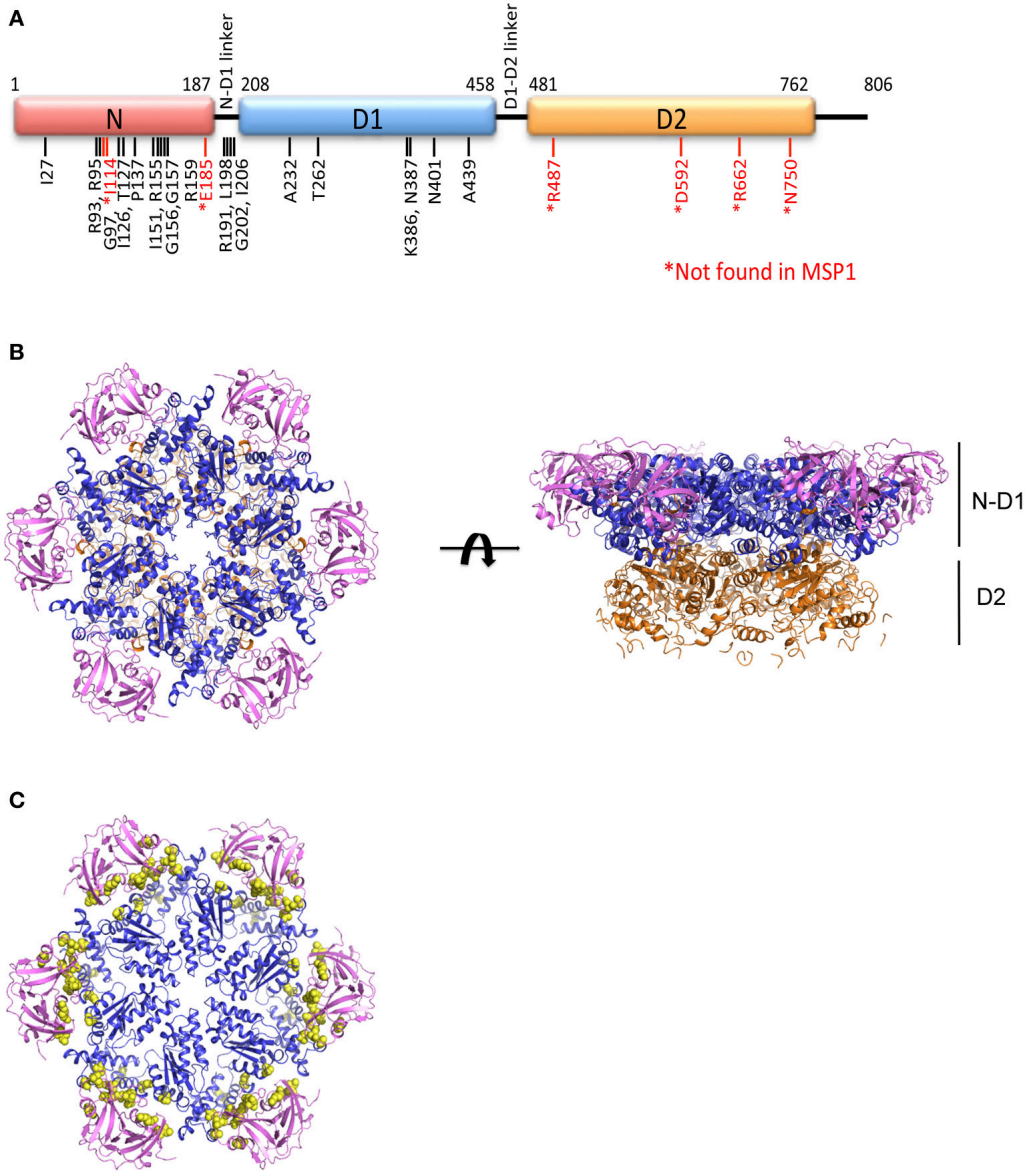
**Structure of the AAA ATPase p97. (A)** Schematic domain organization of a p97 subunit showing the three structural domains: N-terminal N domain and two ATPase domains D1 and D2, and the positions of pathogenic mutations. **(B)** Ribbon representation of the top and side views of the hexameric structure of ^FL^p97 (PDB:3CF2, Davies et al., 2008). The N domain is in purple, D1 domain in blue and D2 domain in gold. **(C)** The top view of ^ND1^p97 structure showing the location of pathogenic mutations. Selected pathogenic mutations (residue I27, R93, I126, P137, R155, R191, L198, I206, A232, T262, N387, N401, A439) are represented as yellow spheres on the ribbon diagram of ^ND1^p97 with ADP bound (PDB: 1E32, Zhang et al., 2000).

MSP1 (OMIM #167320, also called *Inclusion bodies myopathy with Paget's disease of bone and frontotemporal dementia, IBMPFD*) is an autosomal dominant disorder, meaning a single copy of the altered gene from either parent is sufficient to cause the disease. There are also cases of new mutations occurring in individuals with no family history of the disorder. The disease is traced to mutations in the gene that encodes p97, also known as VCP (valosin-containing protein) (Kimonis et al., 2000). MSP1 can affect multiple tissues including muscles, bones, and brain (Benatar et al., 2013; Kim et al., 2013). The first symptom of the disease is often muscle weakness (IBM, inclusion body myopathy), which typically appears late in life when the patient is at the age of 50–60 years old, and is found in more than 90% of cases. Half of the cases develop Paget's disease of the bone (PD), which interferes with the recycling process of new bone tissue replacing old one, causing abnormal bone formation. Bone pain, particularly in the hips and spine, is common. One-third of the cases also involve a brain condition called frontotemporal dementia (FTD). This disorder progressively damages parts of the brain that control reasoning, personality, social skills, speech and language, leading to personality changes, a loss of judgment and inappropriate social behavior. So far, more than 20 missense amino acid substitutions on p97 have been identified in MSP1 patients, all located in the N-terminal and D1 domains of the protein and none is found in the D2 domain (Figure 1A and Table 1).

**Table 1:** **Pathogenic mutations in p97**.

	**Change in amino acid**	**Change in gene**	**Location in protein**	**Phenotype**	**References**
I27	I27V	79A>G	N domain	IBM, FTD, PDB	Rohrer et al., 2011; Majounie et al., 2012; Weihl et al., 2015
R93	R93C	277C>T	N domain	IBM, PDB, FTD	Guyant-Maréchal et al., 2006; Hübbers et al., 2007
	R93H	278G>A	N domain	HSP	Neveling et al., 2013
R95	R95C	283C>T	N domain	IBM, ALS	Weihl et al., 2015
	R95H	284G>A	N domain	AD	Kaleem et al., 2007
	R95G	283C>G	N domain	IBM, PDB, FTD, ALS	Watts et al., 2004; Kimonis et al., 2008b
G97	G97E	290G>A	N domain	IBM, PDB, FTD	Gu et al., 2012; Jerath et al., 2015
I114	I114V	340A>G	N domain	ALS	Koppers et al., 2012
I126	I126F	376A>T	N domain	IBM, PDB, FTD	Matsubara et al., 2016
T127	T127A	379A>G	N domain	FTD, AD	Shi et al., 2016
P137	P137L	410C>T	N domain	IBM, PDB, FTD	Stojkovic et al., 2009; Palmio et al., 2011
I151	I151V	451A>G	N domain	IBM, ALS	DeJesus-Hernandez et al., 2011; Boland-Freitas et al., 2016
R155	R155S	463C>A	N domain	IBM, PDB, FTD	Stojkovic et al., 2009
	R155L	464G>T	N domain	IBM, PDB, FTD	Kumar et al., 2010
	R155H	464G>A	N domain	IBM, PDB, FTD, ALS	Watts et al., 2004; Hübbers et al., 2007; Kimonis et al., 2008a; Viassolo et al., 2008; Stojkovic et al., 2009; González-Pérez et al., 2012
	R155C	463C>T	N domain	IBM, PDB, FTD, ALS	Watts et al., 2004; Schröder et al., 2005; Guyant-Maréchal et al., 2006; Gidaro et al., 2008; González-Pérez et al., 2012
	R155P	464G>C	N domain	IBM, PDB, FTD	Watts et al., 2004
G156	G156C	466G>C	N domain	ALS	Segawa et al., 2015
	G156S	466G>A	N domain	IBM, PDB, FTD	Komatsu et al., 2013
G157	G157R	469G>C	N domain	IBM, PDB, FTD	Djamshidian et al., 2009
		469G>A	N domain	IBM, PDB, FTD	Stojkovic et al., 2009
M158	M158V	472A>G	N domain	PDB, ALS	Ayaki et al., 2014
R159	R159G	475C>G	N domain	ALS, FTD	Johnson et al., 2010
	R159C	475C>T	N domain	IBM, FTD, PD, ALS	Bersano et al., 2009; Chan et al., 2012; de Bot et al., 2012; González-Pérez et al., 2012
	R159H	476G>A	N domain	IBM, PDB, FTD, ALS	Haubenberger et al., 2005; Stojkovic et al., 2009; van der Zee et al., 2009; Koppers et al., 2012
E185	E185K	553C>T	N domain	CMT2Y	Gonzalez et al., 2014
R191	R191G	571C>G	N-D1 linker	IBM, ALS	González-Pérez et al., 2012
	R191Q	572G>A	N-D1 linker	IBM, PDB, FTD, ALS	Watts et al., 2004; Kimonis et al., 2008b; Stojkovic et al., 2009; Johnson et al., 2010; González-Pérez et al., 2012
L198	L198W	593T>G	N-D1 linker	IBM, PDB, FTD	Watts et al., 2007; Kumar et al., 2010
G202	G202W	604G>T	N-D1 linker	IBM, FTD	Figueroa-Bonaparte et al., 2016
I206	I206F	616A>T	N-D1 linker	IBM, PDB, FTD	Peyer et al., 2013
A232	A232E	695C>A	D1 domain	IBM, PDB	Watts et al., 2004; Kimonis et al., 2008b
T262	T262A	784A>G	D1 domain	IBM, PDB, FTD	Spina et al., 2008
K386	K386E	1158T>C	D1 domain	IBM	Lévesque et al., 2016
N387	N387H	1159A>C	D1 domain	IBM, FTD	Watts et al., 2007
	N387S	1160A>G	D1 domain	IBM, PDB, FTD	Liewluck et al., 2014
	N387T	1160A>C	D1 domain	ALS	Abramzon et al., 2012
N401	N401S	1202A>G	D1 domain	FTD, ALS	Shi et al., 2016
A439	A439S	1315G>T	D1 domain	IBM, PDB	Stojkovic et al., 2009
	A439P	1315G>C	D1 domain	IBM, PDB, FTD	Shi et al., 2012; Kamiyama et al., 2013
	A439G	1316C>G	D1 domain	IBM, FTD	Figueroa-Bonaparte et al., 2016
R487	R487H	1460G>A	D2 domain	FTD, ALS	Hirano et al., 2015
D592	D592N	1774G>A	D2 domain	ALS	Johnson et al., 2010
R662	R662C	1984C>T	D2 domain	ALS	Abramzon et al., 2012
N750	N750S	2249A>G	D2 domain	ALS	Kenna et al., 2013

*IBM, inclusion body myopathy; PDB, Paget's disease of bone; FTD, frontotemporal dementia; PD, Parkinson disease; ALS, amyotrophic lateral sclerosis; CMT2Y, Charcot-Marie-Tooth disease*.

### Familial amyotrophic lateral sclerosis (FALS)

ALS or Lou Gehrig's disease is a progressive neurodegenerative disease that affects the motor neurons in the brain and spinal cord. When these nerve cells die, the brain loses the ability to control muscle movement, causing complete paralysis in late stages of the disease and eventually death. In about 90% of cases, the cause of ALS is sporadic, which means they are not inherited. Pathological hallmarks of ALS are pallor of corticospinal tract due to loss of motor neurons, the presence of ubiquitin-positive inclusions and the deposition of pathological TDP-43 aggregates. The cause of this sporadic ALS is not well understood; it may be due to a combination of environmental and genetic risk factors. About 10% of cases are considered “familial ALS” (FALS, OMIM #613954). In these cases, more than one individual in the family develops ALS and sometimes family members have FTD as well. Mutations in at least 18 genes have been identified in FALS cases, with mutations in the p97 gene contributing <1–2% (Table 1) (Johnson et al., 2010; Koppers et al., 2012; Kwok et al., 2015).

### Charcot-marie-tooth disease, type 2Y (CMT2Y)

CMT2Y (OMIM #616687) is an autosomal dominant axonal peripheral neuropathy characterized by distal muscle weakness and atrophy associated with length-dependent sensory loss. The disease CMT is named after the three physicians who first accurately described it in 1886: Jean-Martin Charcot and Pierre Maries in France, and Howard Henry Tooth in England. Its principal features include slowly progressive muscular atrophy, which initially involves the feet and legs, but does not affect the upper extremities until several years later. CMT is a clinically and genetically heterogeneous disorder and is divided into subtypes based on genetics, pathology, and electrophysiology of the disease (Dyck and Lambert, 1968). The subtype CMT2Y has missense mutations in the p97 gene, which were identified in patients (Gonzalez et al., 2014; Jerath et al., 2015) (Table 1). As most patients with CMT2Y do not obtain a genetic diagnosis, the number of cases having mutations in p97 may be higher than expected.

## Structural and biochemical differences between wild-type and pathogenic p97

### Structure of p97

P97 is a Type II AAA^+^ ATPase (two AAA ATPase domains) and a homo-hexamer with each subunit consisting of three main domains: the N-terminal domain (N domain) followed by two tandem ATPase domains (D1 and D2 domains), which are connected by two short polypeptides (N-D1 and D1-D2 linker). Both the D1 and D2 domains possess all essential sequence elements (Walker A and B motifs) for ATP hydrolysis and share high amino acid sequence identity. The N domains are known for interacting with various cofactors and adaptor proteins. Cofactors of p97 are defined as those proteins that are necessary for p97 function, whereas adaptors are those that target p97 to different cellular locations (Xia et al., 2016). At first glance, a p97 hexamer appears to have two rings of different sizes stacked on top of each other. The crystal structure of full-length wild-type p97 (^FL^p97) reveals that the two ATPase domains form two concentric rings, called D1 and D2 rings, and the N domains are attached to the periphery of the D1 ring (DeLaBarre and Brunger, 2003) (Figure 1B). The hexameric architecture of p97 is maintained by interactions among the D1 domains (Wang et al., 2003), as isolated D2 domains are prone to form heptamers (Davies et al., 2008). This hexameric structure of p97 is very stable and can withstand treatment of up to 6M urea and its assembly does not require the addition of nucleotide (Wang et al., 2003).

More than 20 amino acid mutations have been identified in p97 from MSP1 or IBMPFD patients and these mutations appear to be randomly scattered throughout the sequence of the N and D1 domain of p97 (Figure 1A). However, when mapped to the structure of ^FL^p97, these MSP1 mutations were found exclusively at the interface between the N and D1 domain (Figure 1C). None was found at the sites where ATP hydrolysis occurs. Structural studies using X-ray crystallography show the pathogenic mutants retain a hexameric ring structure and share identical overall folding with the wild-type protein (Tang et al., 2010).

### Amount of pre-bound ADP

One important characteristic of p97 related to binding of nucleotides is the presence of pre-bound ADP at the D1 domain, which was hinted at by p97 crystallization experiments in the presence of different types of nucleotides. Crystallographic efforts with wild-type p97 yielded ADP invariably bound to the D1 domain, while various types of nucleotides bound to the D2 domain (Zhang et al., 2000; DeLaBarre and Brunger, 2003), leading to the misconception that the D1 domain was incapable of exchanging for different types of nucleotides. Subsequent experiments led to the realization that the nucleotide state at the D1 domain of p97 is tightly regulated (Davies et al., 2005). Without the addition of any ADP during the course of purification, isolated wild-type p97 was shown to have tightly bound ADP at the D1 domain with at least 3 molecules of ADP per p97 hexamer (DeLaBarre and Brunger, 2003; Briggs et al., 2008; Tang and Xia, 2013). This phenomenon is referred to as the pre-bound ADP at the D1 domain. Apparently, a subset of D1 domains in the hexameric p97 is occupied by ADP, thus preventing saturation of all D1 sites with ATP, which has a higher binding affinity for an empty D1 site (Tang et al., 2010; Tang and Xia, 2013). Thus, structural studies of the conformational change of wild-type p97, especially at low resolution where the nucleotide state is uncertain, should take the feature of the pre-bound ADP into account when interpreting the results.

Compared with wild-type p97, pathogenic mutants have less pre-bound ADP (Tang and Xia, 2013). More importantly, these mutants are not able to tightly regulate the nucleotide state of the D1 domain, as does the wild-type p97. They allow ATP to displace pre-bound ADP. Consequently, a uniform binding of ATP to the D1 sites can be observed (Tang et al., 2010; Tang and Xia, 2013).

### Communication among domains and subunits

In each biological unit of p97, there are six identical subunits, containing a total of 18 main domains. The proper function of p97 therefore relies on a coordinated interplay among these domains. For instance, the conformation of the N domain has a strong influence over the ATPase activity of p97. Fixing the N domain position by introducing a disulfide bond between the N and the D1 domain reduces p97 ATPase activity (Niwa et al., 2012). The binding of adaptor proteins such as p47 and p37 to the N domain alter the overall ATPase activity of p97 (Meyer et al., 1998; Zhang et al., 2015). On the other hand, the nucleotide states of the D1 domains control the conformations of the N domain of p97 (Tang et al., 2010; Banerjee et al., 2016; Schuller et al., 2016).

The binding of ATP in the D1 domain is required for the activity of the D2 domain, and vice versa (Ye et al., 2003; Nishikori et al., 2011; Tang and Xia, 2013). One of the possible mechanisms of communication between these two ATPase domains is through the D1-D2 linker. This 22-residue linker peptide contains a highly conserved N-terminal half that appears to be a random loop and extends to the vicinity of both the D1 and D2 nucleotide-binding sites, as illustrated in the ^FL^p97 structures (Davies et al., 2008). The inclusion of the D1-D2 linker to the N-D1 truncate of p97 activates the ATPase activity of the D1 domain (Chou et al., 2014; Tang and Xia, 2016).

Among the three domains of a p97 subunit, the D1 domain seems to play a role consistent with (1) maintaining the hexameric architecture of p97 (Wang et al., 2003), (2) driving the conformational change of the N domain (Tang et al., 2010; Banerjee et al., 2016; Schuller et al., 2016), (3) regulating the activity of the D2 domain (Tang and Xia, 2013), and (4) communicating with and controlling the nucleotide states of D1 domains of neighboring subunits (Tang and Xia, 2013, 2016; Zhang et al., 2015). All these suggest an intricate communication network centered on the D1 ring of the hexameric p97.

Instead of causing structural changes to the protein, pathogenic p97 mutations appear to alter the function of p97 by perturbing the communication network between domains. Our experiments have shown that while the domain communication within an individual subunit remains undisturbed, communication between neighboring subunits in pathogenic mutants has changed, leading to uncoordinated nucleotide binding among different subunits (Tang et al., 2010; Tang and Xia, 2013). Specifically, the mutations weaken the ADP-binding affinity at the D1 domain and thus relax the tight regulation of the nucleotide states at the D1 domains (Tang and Xia, 2013). As a result, more ATPase domains of mutants are engaged in ATP hydrolysis compared to wild-type p97, giving rise to an apparent more active protein with higher ATPase activity (Halawani et al., 2009; Manno et al., 2010; Tang et al., 2010; Niwa et al., 2012).

### Nucleotide-driven conformational changes

It is generally believed that p97 functions as a molecular extractor, pulling damaged or unwanted proteins from large molecular or cellular assemblies. It does so by undergoing ATP-dependent conformational changes to generate mechanical forces necessary for substrate extraction (Acharya et al., 1995; Latterich et al., 1995; Rabouille et al., 1995; Xu et al., 2011; Ramanathan and Ye, 2012; Xia et al., 2016). Although exactly how p97 extracts substrate from a large molecular assembly remains unclear, progress has been made in identifying different conformations. Low-resolution cryo-EM studies showed a moderate rotational movement between the D1 and D2 rings in association with changes in the size of the D2 central pore in response to the presence of different nucleotide (Rouiller et al., 2002). However, a similar study by another group suggested a different domain movement (Beuron et al., 2003). The insufficient resolution to determine the exact nucleotide state in each domain of p97 in these studies could be the cause of the inconsistency.

**Figure 2:**
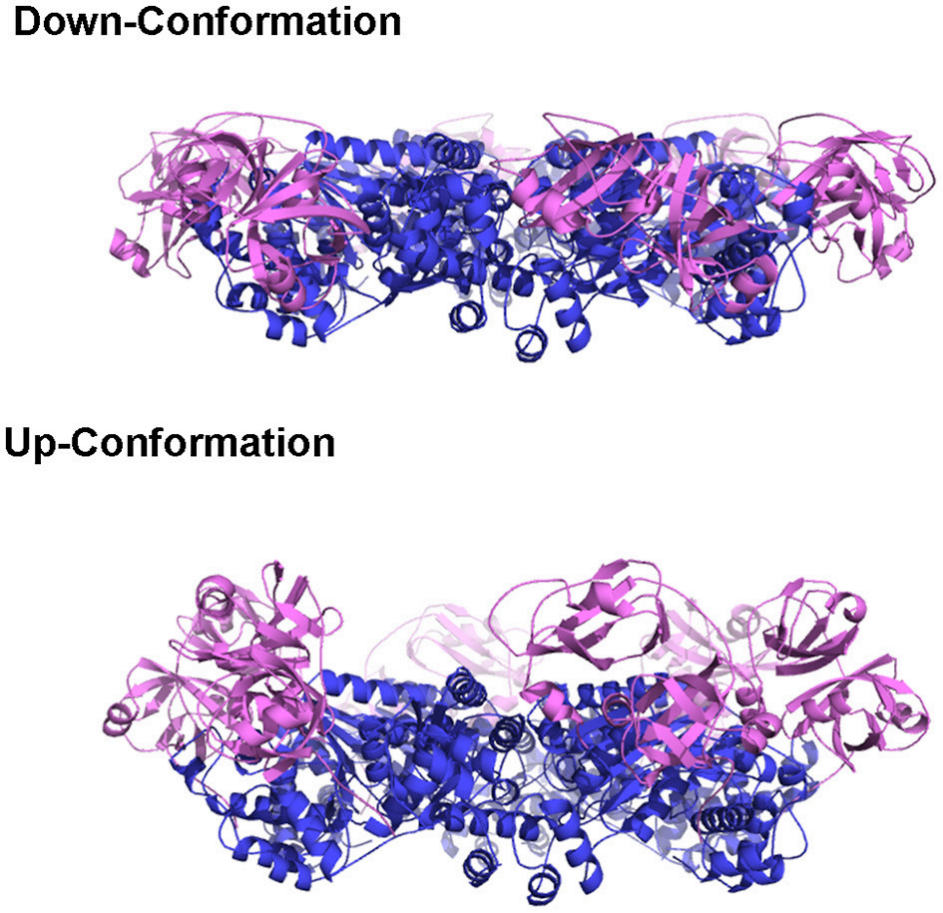
**The Up- and Down-conformation of p97 N domain**. Ribbon presentation of the structure of the hexameric ^ND1^p97. The D1 domains are colored in blue and the N domains are in purple.

Earlier crystallographic studies showed the D1 domains are always bound with ADP, regardless of the presence of different types of nucleotides in solution, and the N domains are in a conformation that is coplanar with the D1 ring (Zhang et al., 2000; DeLaBarre and Brunger, 2003; Davies et al., 2008). This N domain conformation when the D1 domain is occupied with ADP is termed the Down-conformation (Figure 2) (Tang et al., 2010). On the other hand, the nucleotide-binding state in the D2 domains is determined by what is present in solution (either bound ADP, AMP-PNP, or ADP-AlFx). Therefore, these crystallographic data can only reveal the conformational changes associated with the nucleotide state at the D2 domain. The D2 ring undergoes a rotation relative to the D1 ring and size of the D2 central pore changes during ATP cycle, but whether the binding or the hydrolysis of ATP triggers the opening remains controversial (Davies et al., 2005; Pye et al., 2006; Banerjee et al., 2016; Hänzelmann and Schindelin, 2016b; Schuller et al., 2016). It is worth pointing out that, for the same nucleotide state, non-uniform domain conformation is observed in subunits within a crystallographic asymmetric unit, and the magnitude of such a difference is comparable to that observed between different nucleotide states (Davies et al., 2008). It is unclear if the conformational differences observed in various nucleotide states of the D2 domain represent actual changes in solution.

Recently, by genetically modifying some regions in the D2 domain, Hanzelmann and colleagues were able to determine the crystal structure of full-length p97 with both ATPase domains either empty or bound with ATPγS (non-hydrolyzing ATP analog) (Hänzelmann and Schindelin, 2016b). The binding of ATPγS opens the D2 pore and generates a rotational movement between the two concentric rings. However, questions remain concerning the physiological relevance of these observations, as the effect of these mutations on the function of p97 was not characterized.

Pathogenic mutations weaken the ADP binding interactions at D1 sites and alter the regulation imposed among neighboring subunits. Effects of these mutations, though very subtle, are sufficient to make these mutants achieve uniform N domain conformation or loss of asymmetry within the hexamer, which is a property that facilitates crystallographic studies. When ATPγS binds to the D1 sites of the N-D1 fragment of p97, the N domains move to a position above the D1 ring, which is termed the Up-conformation (Figure 2) (Tang et al., 2010). Such nucleotide-dependent conformational switch has also been detected for only a subset of subunits in wild-type p97 in solution (Tang et al., 2010). The nucleotide-dependent conformational movement of the N domain has been confirmed by recent studies of full-length wild-type p97 using single particle cryo-EM (Banerjee et al., 2016; Schuller et al., 2016). Instead of having all six p97 subunits in the Up-conformation in the presence of ATPγS or AMP-PMP, Schuller and colleagues observed a distribution of N domain conformations, either in Up- or Down-conformation within a hexamer (Schuller et al., 2016). By contrast, Benerjee and colleagues only reported a single conformation that N domains of all subunits were in the Up-conformation, despite the very weak EM density for the N domain (Banerjee et al., 2016). More interestingly, crystal structure of the full-length p97 with genetically modified D2 domain showed the N domain remains in the Down-conformation when the D1 domain is bound with ATPγS (Hänzelmann and Schindelin, 2016b). Thus, whether the six nucleotide-binding sites in the D1 ring bind ATP in a concerted manner leading to symmetrical N domain movement or in a sequential/random manner leading to asymmetrical hexamer has yet to come to a consensus. However, the presence of tightly pre-bound ADP in the D1 domains of a subset of p97 subunits may have already suggested a non-uniform nucleotide binding of p97.

**Figure 3:**
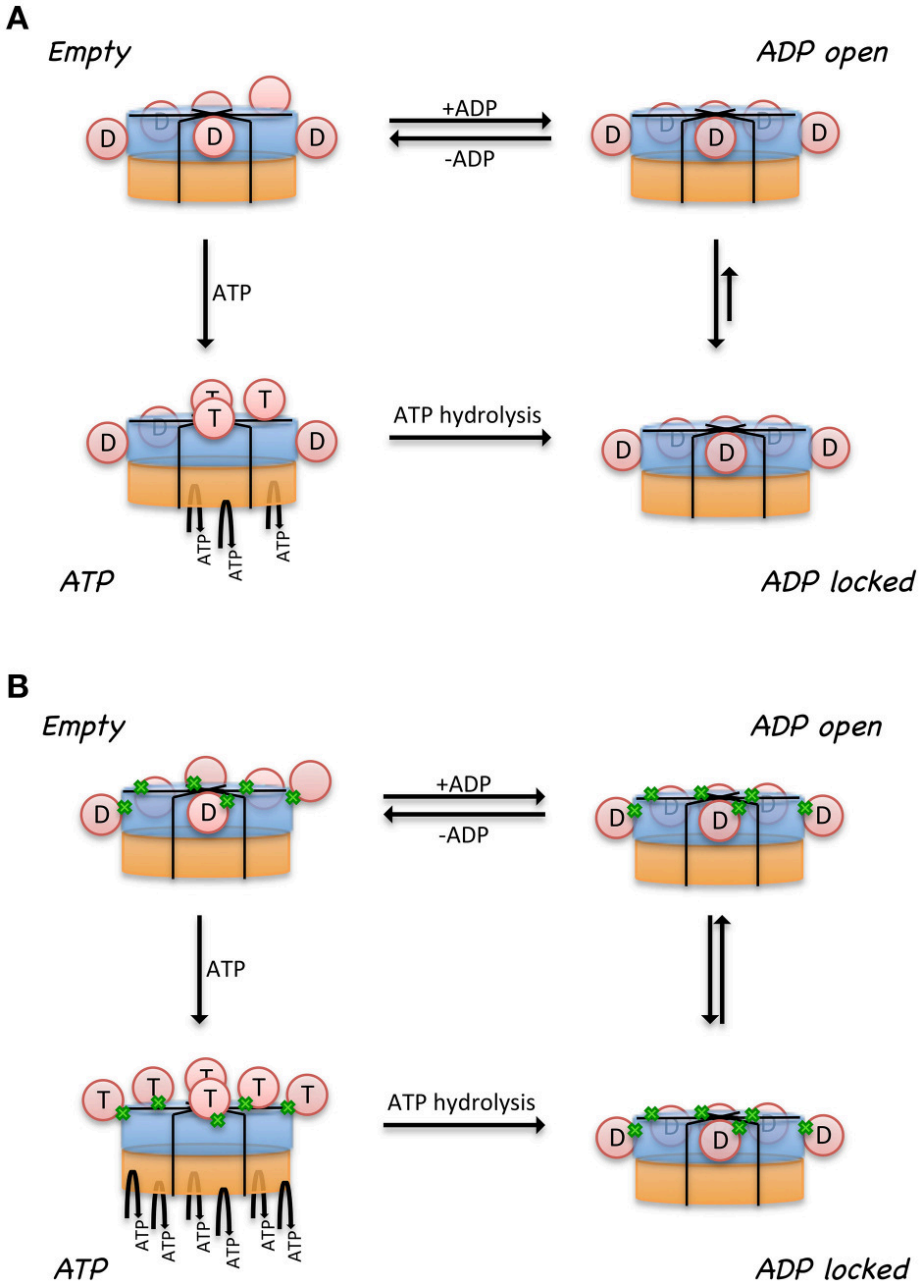
**Model proposed for the relationship between control of N domain conformation and ATPase activity in p97**. Cartoon representation of a hexameric p97 shows the D1 domains in blue, D2 domains in orange, and the N domains in pink circle. N domains that are labeled with the letter “D” are in the Down-conformation and their corresponding D1 domains are occupied by ADP. N domains that are labeled with the letter “T” are in the Up-conformation and their corresponding D1 domains are occupied with ATP. Only those subunits that have their D1 domains occupied by ATP are capable of hydrolyzing ATP in their D2 domains. The conformation of the N domains is not determined when their corresponding D1 domains are empty (No label in the N domain). Proposed nucleotide binding and hydrolysis cycle for **(A)** the wild-type p97 and for **(B)** mutant p97. Mutations are represented by the green dots at the interface between N and D1 domains. Empty state indicates a state in the absence of added ATP or ADP. The ATP state is the presence of added ATP. The ADP-locked state refers to a subset of subunits where ADP in the D1 domain is very tightly bound, whereas the ADP-open state refers to a subset of subunits where ADP molecules in the D1 domains are able to exchange nucleotides with those in solution. The ADP-locked and ADP-open states are in equilibrium in solution.

A model was proposed to illustrate the regulatory mechanism of ATP binding and hydrolysis in the D1-ring and how it might influence the ATPase activity of the D2 ring (Figure 3A) (Tang et al., 2010; Tang and Xia, 2013). In this model, there are four states for a subunit of a wild-type p97 hexamer, each representing one specific nucleotide-binding state. (1) There is an Empty state where no nucleotide is bound at the D1 site; the conformation of the N- domain is unknown (pink sphere). Noticed that for a wild-type p97 hexamer, only a subset of subunits is in the Empty state because of the pre-bound ADP. The N domains for those with pre-bound ADP are in the Down-conformation and are shown as pink sphere labeled with D. (2) When ATP enters the D1 site (ATP state), it is only allowed in the Empty subunits and not allowed in those with pre-bound ADP. The subunits with ATP bound have their N domain adopt the Up-conformation (pink sphere labeled with T), which has been determined from the crystal structure of IBMPFD mutants (Tang et al., 2010). (3) The hydrolysis of ATP to ADP at the D1 domain brings the N domain back to the Down-conformation, which is supported by the crystallographic data from both wild-type p97 and IBMPFD mutants (Zhang et al., 2000; DeLaBarre and Brunger, 2003; Huyton et al., 2003; Tang et al., 2010). (4) Importantly, it was proposed that there are two ADP-bound states existing in equilibrium for a subunit: an ADP-locked and ADP-open state. Both ADP-open and ADP-locked states can coexist for different subunits in a p97 hexamer. The ADP-locked state is inspired by the presence of pre-bound ADP at the D1 site in the wild-type p97, which is difficult to remove (Davies et al., 2005; Briggs et al., 2008; Tang et al., 2010). The ADP-open state represents the situation where ADP has a reduced affinity to the D1 site ready to be exchanged. (5) It was also proposed that the D2 domain of a subunit is permitted to hydrolyze ATP only if its cognate D1 domain is occupied by ATP.

A major difference between the wild-type and mutant p97 was proposed to be the regulation of the inter-conversion or the equilibration between the ADP-open and ADP-locked state (Figure 3B). In the wild type, the equilibration favors the ADP-locked state, whereas in the mutant, it prefers the ADP-open state. This means, in the case of a wild-type p97 hexamer, that ATP can only get into a subset of D1 domains, driving corresponding N domains to the Up-conformation. This non-uniform nucleotide-binding state in the wild-type p97 in the presence of ATP generates an asymmetry in the N domain conformation in a hexameric p97. In p97 mutants, the equilibration between ADP-locked and ADP-open states is shifted toward the latter. As a result, a uniform nucleotide-binding state at the D1 domains and a synchronized N domain movement can be reached in the presence of a sufficiently high concentration of ATP, forming symmetrical hexamers. More importantly, this model implies that the function of p97 requires an asymmetry in the D1 nucleotide-binding state in a hexameric ring. We should also point out that a consequence of this model is that the p97 mutants are higher in ATPase activity, because there are more ATP molecules occupying the D1 sites, which is required for ATP hydrolysis in the D2 domain (Tang and Xia, 2013).

Although the role of the conformational changes observed in p97 during the ATP cycle in relation to its physiological function remains unclear, the opening and closing of the D2 pore as well as the up-and-down swinging motion of the N domain have consistently been observed. As experimental evidence increasingly points to a role played by p97 in extracting protein substrates from its interacting partners, the coordinated up-and-down motion of the N domain at the D1 ring and the opening and closing of the D2 ring within the hexamer during the ATP hydrolysis could conceivably generate a pulling force to extract protein substrates from various organelles. Taking ERAD as an example, p97 is recruited to the ER membrane via interaction between the N domain and adaptor proteins. The swinging movement of the N domain would create a pulling force to extract the protein substrates from the ER membrane. Conceivably, the generation of this pulling force requires a highly sophisticated coordination among the subunits of p97. As shown from biochemical and structural studies, individual subunits of pathogenic mutants fail to communicate, resulting in uniform movement of the N domain. This un-coordinated conformational change in pathogenic p97 may be why mutants fail to process protein substrates effectively, thus leading to accumulation of protein inclusions.

### Interacting with protein partners

Over 30 different cofactor/adaptor proteins have been identified; they interact mostly with the N domain but in some cases the C-terminal tail of p97. These proteins either function as adaptors that recruit p97 to a specific subcellular compartment or substrate, or serve as cofactors that help in substrate processing. They are found in many different subcellular structures such as mitochondria, endoplasmic reticulum membrane, nuclear membrane, and Golgi body. Hence, their bindings lead p97 to function in different cellular pathways.

**Figure 4:**
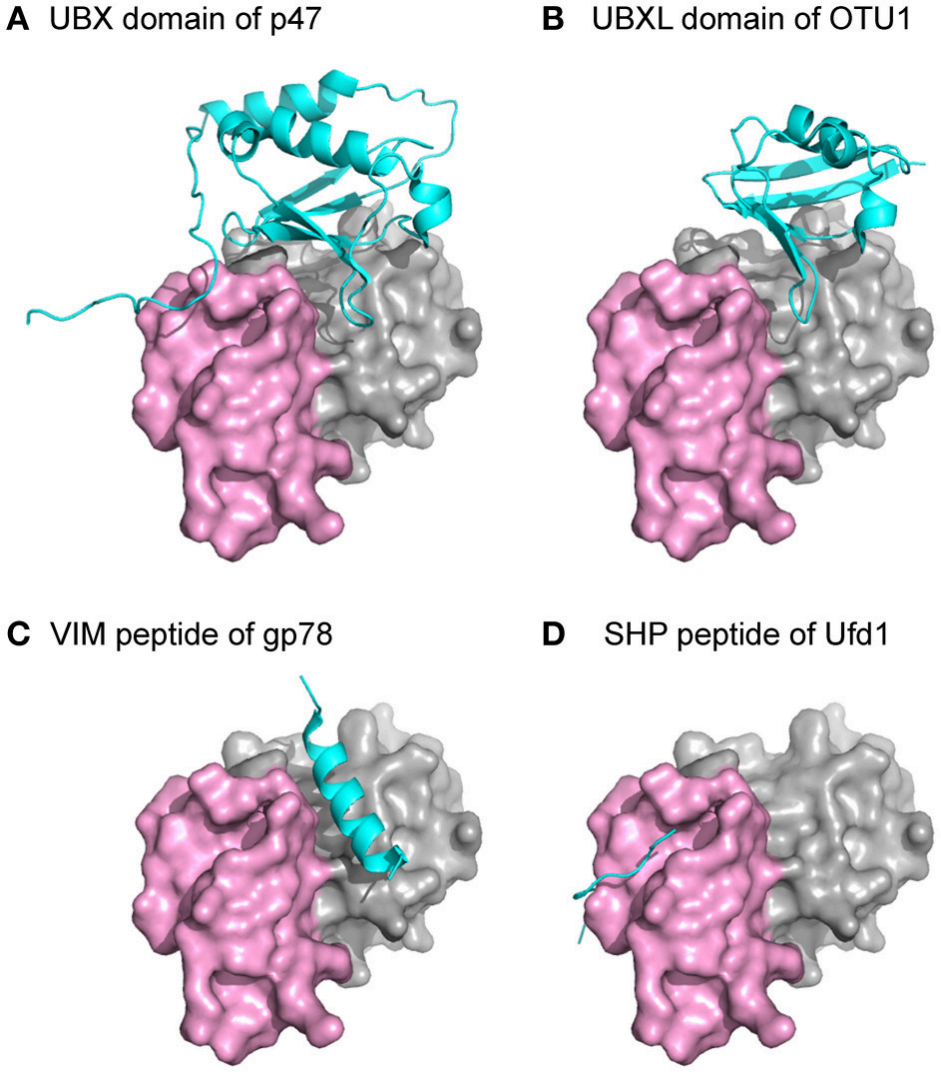
**Structures of p97 in complex with interacting proteins**. The N domain of p97 is shown as a surface representation with the two subdomains, double ψ-barrel and β-barrel, in gray and violet, respectively. Individual domains or peptides from different p97-interacting proteins are shown as a cyan cartoon. All the structures were superposed with the N domain of p97 and presented in the same orientation. **(A)** PDB:1S3S (Dreveny et al., 2004). **(B)** PDB:4KDI (Kim et al., 2014). **(C)** PDB:3TIW (Hänzelmann and Schindelin, 2011). **(D)** PDB:5C1B (Hänzelmann and Schindelin, 2016a).

Several common binding-domains or motifs, such as the UBX domain, the PUB-domain, and the VCP-interacting motif (VIM), have been found to interact with p97. Despite differences in structures among these binding motifs, most of them bind to the N domain at the interface between the two subdomains, as shown from crystal structures of these binary complexes (Figure 4). This observation provides an explanation for the mutually exclusive binding pattern observed biochemically among various p97-interacting proteins (Meyer et al., 2000; Rumpf and Jentsch, 2006). Intriguingly, while all six binding interfaces on the N domains of a hexameric p97 are available, crystal structures of the complexes showed the binding stoichiometry is not more than 3 molecules of adaptor proteins to 1 ^FL^p97 hexamer (Dreveny et al., 2004; Hänzelmann and Schindelin, 2016a). Consistently, binding studies using the isothermal calorimetry (ITC) technique showed a similar effect (Hänzelmann et al., 2011). Indeed, the sharing of the same binding interface and the substoichiometric binding of the interacting protein to p97 led to the hierarchical binding model for p97 to fulfill specific cellular functions (Hänzelmann et al., 2011; Meyer et al., 2012).

The impact of pathogenic mutations on the interactions between p97 and adaptor proteins has been investigated. So far, there is no structural data in the literature that demonstrate the difference in adaptor protein binding between wild-type and mutant p97. Using isolated ^FL^p97, it was shown biochemically that cofactors p37 and p47 regulate ATPase activity of p97 in a concentration-dependent manner. By contrast, mutant p97 lost this regulation although it still interacts with the cofactors (Zhang et al., 2015). Results derived from cell-based experiments from different groups are not always consistent (Fernández-Sáiz and Buchberger, 2010; Manno et al., 2010). For example, in one study, isolated mutant p97 exhibited the same binding as wild-type p97 toward the adaptor proteins p47, Ufd1-Npl4, and E4B, the human UFD-2 homolog. However, mutants in the same study showed impaired binding to ubiquitin ligase E4B in the presence of Ufd1-Npl4. *In vivo* pull-down experiments using HEK293 cells showed reduced binding toward the E4B and enhanced binding toward ataxin 3, thus resembling the accumulation of mutant ataxin 3 on p97 in spinocerebellar ataxia type 3 (Fernández-Sáiz and Buchberger, 2010). In another study, however, similar *in vivo* pull-down were carried out showing enhanced binding of the Ufd1-Npl4 pair by IBMPFD mutants but not for p47 (Manno et al., 2010). An increased amount of cofactor pair Ufd1-Npl4 was detected in association with mutant p97 (Fernández-Sáiz and Buchberger, 2010; Manno et al., 2010). However, no significant difference was found in the binding of the same adaptor to either wild-type or pathogenic mutants when using isolated protein for pull-down assays (Hübbers et al., 2007; Fernández-Sáiz and Buchberger, 2010). This inconsistency may be due to the difference in the N domain conformation, which depends on the nucleotide state at the D1 domain of p97. Such an effect can be demonstrated by the seven-fold decrease in the binding affinity of SVIP to pathogenic p97 in the presence of ATPγS (Hänzelmann et al., 2011). So far, two nucleotide-dependent conformations (the Up- and Down-conformation) of the N domain have been observed in p97. In both cases, the binding interface for adaptor proteins is available but orients differently. In the Up-conformation, the binding interface faces outward to the side of the hexameric ring, while in Down-conformation, the binding interface faces down toward the D2 ring. As the sizes and shapes of adaptor proteins vary, it is conceivable that the binding of some adaptor proteins will be hindered by spatial restrictions caused by different N domain conformations.

## Functional defects in pathogenic p97

The diverse biological roles played by p97 in various cellular activities, such as membrane fusion, DNA repair, and protein homeostasis, have been reported and extensively reviewed (Dantuma and Hoppe, 2012; Meyer et al., 2012; Yamanaka et al., 2012; Franz et al., 2014; Meyer and Weihl, 2014; Xia et al., 2016). These important functional roles are reflected by the sequence conservation of the protein and indicate that mutations in p97 would have severe functional consequences. Despite embryonic lethality in p97 knock-out mice (Müller et al., 2007) and accelerated MSP1 pathology in homozygote p97 mutant mice (Nalbandian et al., 2012), pathogenic mutations in p97 seems well tolerated and affect only a subset of its functions, as there is no evidence of developmental abnormalities in affected individuals (Kimonis et al., 2008b). This is consistent with the fact that MSP1 is a late-onset disease and clinical pathology of MSP1 seems to point to a defective function in maintaining protein homeostasis.

Pathological features in MSP1 patient samples include rimmed vacuoles found in muscle tissues that stain positive for p97 and ubiquitin (Watts et al., 2004) and nuclear inclusions in neurons, which also stained positive for p97 and polyubiquitin in brain tissues (Kimonis and Watts, 2005; Schröder et al., 2005). This common pathologic feature found in MSP1 affected tissues suggests a defective function of pathogenic p97 mutants in protein degradation/trafficking pathways. Similar phenotypes can be reproduced in *in vitro* cultured cells, either transfected with disease-associated p97 mutants (Weihl et al., 2006; Janiesch et al., 2007) or derived from patient tissues (Ritz et al., 2011). Moreover, studies using various animal models further strengthen the linkage between mutations in p97 and MSP1. Transgenic mice bearing a p97 mutation (R155H or A232E) display dominant-negative phenotypes similar to MSP1 patients (Weihl et al., 2007; Custer et al., 2010); mutant p97 (R155H) knock-in mice display progressive muscle weakness and other MSP1-like symptoms (Badadani et al., 2010).

One of the best studied cellular functions of p97 is endoplasmic reticulum-associated degradation (ERAD) (Meyer et al., 2012). Protein substrates in the ER are labeled with polyubiquitin chains, recognized, and subsequently retrotranslocated by p97 across the ER membrane to the cytosol, where they are degraded by the proteasome. Failure to clear these polyubiquitinated protein substrates leads to ER stress. It has been shown that MSP1 mutants have impaired ERAD, leading to accumulation of ERAD substrates (Weihl et al., 2006; Erzurumlu et al., 2013).

Another characteristic that sets pathogenic mutants apart from wild-type p97 is their failure to form a ternary complex with ubiquitylated CAV1 (Ritz et al., 2011). CAV1 (caveolin-1) is a main constituent of caveolae, small invaginations on the plasma membrane. The degradation of CAV1 through the endocytic pathway requires mono-ubiquitin modification (Haglund et al., 2003; Parton and Simons, 2007). During maturation, CAV1 first forms SDS-resistant oligomers that associate to form larger assemblies in a cholesterol-dependent manner during exit from the Golgi apparatus. P97 binds to a mono-ubiquitylated cargo substrate, CAV1, on endosomes and is critical for its transport to endolysosomes. Blocking p97 binding of CAV1 with MSP1-associated mutations or its protein segregase activity with the Walker B motif mutation or the DBeQ inhibitor leads to accumulation of CAV1 at the limiting membrane of late endosomes (Ritz et al., 2011).

Besides ubiquitin, TAR DNA-binding protein-43 (TDP-43) is also found in protein inclusions in MSP1 affected tissues (Neumann et al., 2007; Weihl et al., 2008). TDP-43, the major pathological protein in ALS and FTD (Neumann et al., 2006), is primarily localized in the nucleus (Wang et al., 2001) and was suggested to play a role in transcription repression and other cellular processes (reviews please see Wang et al., 2008; Buratti and Baralle, 2009). Although how TDP-43 gets into the protein inclusions in tissue samples of MSP1 patients is unknown, it is believed that TDP-43 is a substrate for either proteasome or autophagic degradation (Caccamo et al., 2009; Wang et al., 2010), hence suggesting a role of p97 in autophagy, a degradation process involving the lysosomal machinery. The role of p97 in autophagy has been demonstrated in both mammalian and yeast cells, in which p97 has been found essential for the maturation of autophagosomes (Tresse et al., 2010). MSP1 mutants have also been observed to accumulate autophagosome markers p62 and LC3-II (Ju et al., 2009; Vesa et al., 2009; Tresse et al., 2010).

## Conclusions and perspective

Since the recognition of the linkage between MSP1 disease and the AAA protein p97 in 2001 (Kovach et al., 2001), there has been a steady increase in the number of pathogenic mutations being identified and increasing number of diseases associated with these mutations in p97. The association of the mutations with the disease calls for a clear understanding of the exact molecular function and its underlying mechanism of p97. Through comparative studies between wild type and mutants and using an array of genetic, biochemical, and structural methodologies, these mutants added a new dimension to our understanding on the structure and function of p97. Despite the progress made, a few fundamental mechanistic questions regarding the action of p97 remain unclear and require further engagement of the research community. First, what is the physiological significance of the conformational changes in p97? To answer this question, an *in vitro* system needs to be established to reconstruct the process identified *in vivo* for p97, which would allow us to investigate the role of p97 in a well-controlled manner and to pinpoint the steps in the reaction coordinates, which are affected by mutations. Secondly, studies are required to further identify properties of p97 that are affected by mutations, such as binding of adaptor/cofactor proteins. Finally, mutations in p97 can cause different diseases. How do cellular factors influence the ultimate clinical outcomes in patients? As a late-onset disease, individuals with p97 mutations can live a normal life for a long time without symptoms. Identifying the factors that delay the onset of the diseases and understanding how they interact with p97 can have a significant impact on those who are predisposed to the disease. The path to address these questions seems unlikely to be straight forward, as pathogenic mutations only manifest their effects in a subtle way and p97 involves in many cellular pathways. Nevertheless, optimism is warranted, given the progresses made in the past, that this path will lead us to the solutions to these unsolved issues.

## Ethics statement

The authors declare no competing financial interests.

## Author contributions

All authors listed, have made substantial, direct and intellectual contribution to the work, and approved it for publication.

### Conflict of interest statement

The authors declare that the research was conducted in the absence of any commercial or financial relationships that could be construed as a potential conflict of interest.
